# Identification of latent tuberculosis infection-related microRNAs in human U937 macrophages expressing *Mycobacterium tuberculosis* Hsp16.3

**DOI:** 10.1186/1471-2180-14-37

**Published:** 2014-02-12

**Authors:** Qing-Lin Meng, Fei Liu, Xing-Yuan Yang, Xiao-Mei Liu, Xia Zhang, Chun Zhang, Zong-De Zhang

**Affiliations:** 1 Suzhou Municipal Key Laboratory of Molecular Diagnostics and Therapeutics, Suzhou Institute of Biomedical Engineering and Technology, Chinese Academy of Sciences, Suzhou 215163, Jiangsu, China; 2 Beijing Key Laboratory of Drug Resistance Tuberculosis, Beijing Tuberculosis and Thoracic Tumor Research Institute, Beijing Chest Hospital, Capital Medical University, Beijing 101149, China; 3 Changchun Institute of Optics, Fine Mechanics and Physics, Chinese Academy of Sciences, Changchun 130033, Jilin, China; 4 College of Life Science and Technology, China Pharmaceutical University, Nanjing 210009, Jiangsu, China

**Keywords:** microRNAs, Macrophages, *Mycobacterium*, *Tuberculosis*, Small heat shock protein, Latent tuberculosis infection

## Abstract

**Background:**

Latent tuberculosis infection (LTBI) relies on a homeostasis of macrophages and *Mycobacterium tuberculosis* (Mtb). The small heat shock protein, Mtb Hsp16.3 (also known as latency-associated antigen), plays an important role in Mtb persistence within macrophages. However, the mechanism of LTBI remains elusive. The aim of this study was to delineate LTBI-related miRNA expression in U937 macrophages expressing Mtb Hsp16.3 protein. U937 macrophages were infected with an integrase-deficient Lentivirus vector to transiently express Mtb Hsp16.3, and green fluorescent protein (GFP) as a control. We used a microRNA (miRNA) microarray chip containing more than 1000 probes to identify the significant differentially expressed miRNAs in the infected U937 cells, and employed real-time quantitative polymerase chain reaction (qRT-PCR) for validation. Furthermore, we confirmed these candidate LTBI-related miRNAs in peripheral blood mononuclear cells from subjects with LTBI and in healthy control individuals. Functional annotation prediction of miRNA target genes and pathway enrichment analyses were used to explore the putative links between these miRNAs and LTBI.

**Results:**

Analysis of the miRNA expression profile identified 149 miRNAs that were differentially expressed in U937 macrophages expressing Mtb Hsp16.3 compared with the control expressing GFP. The expression level of seven miRNAs (miR-424-5p, miR-493-5p, miR-296-5p, miR-27b-3p, miR-377-5p, miR-3680-5p, miR-191-5p) were validated by qRT-PCR. The expression level of four miRNAs (miR-424-5p, miR-27b-3p, miR-377-5p, miR-3680-5p) in the peripheral blood mononuclear cells samples from LTBI and healthy participants reflected the altered patterns observed in the microarray profile. The bioinformatic analyses suggest that the miRNAs may regulate Mtb latent infection by affecting the development of macrophage cells.

**Conclusions:**

The results suggest that miRNA expression may play a considerable role in the pathogenesis of LTBI, and this would increase our understanding of the molecular basis of Hsp16.3-facilitated Mtb survival in macrophages.

## Background

In the latent tuberculosis infection (LTBI) state, the patient harbors the *Mycobacterium tuberculosis* (Mtb) bacilli in the body and is asymptomatic; no radiographic or bacteriological evidence of active tuberculosis is observed; however, the patients reveal immunological sensitization to Mtb-derived antigen proteins (e.g., ESAT6, CFP10, and Hsp16.3) [1]. The granuloma is thought to play a major role in maintaining latency and avoiding reactivation of Mtb, representing the intersection of innate and adaptive immunity. The hypoxic core of the granuloma is thought to induce a dormant state of Mtb. In this regard, *in vitro* studies have confirmed that Mtb dramatically upregulated dormancy survival regulon (DosR)-related genes, which are characteristic of nonreplicating persistence [2]. One of the most prominent of these is Rv2031c, which encodes the small heat shock protein Hsp16.3 (also known as α-crystalline related protein 1, or the 16 kDa antigen). Hsp16.3 constitutes one of the prominent antigens in the exponential phase after infection. It contains both T- and B-cell epitopes that contributes to enhance the cellular and humoral immune responses [3]. Interestingly, Hsp16.3 is maximally expressed during latency, play a role in facilitating the persistence of Mtb within macrophages [4]. Indeed, the functional versatility of macrophages is evident from their role in diverse biological processes, such as phagocytosis, inflammation, immunoregulation, differentiation, and metabolism [5]. Recent studies of these cells using system biology and a variety of -omics technologies in several disease models (e.g., atherosclerosis and metabolic disorders) suggest that they orchestrate crucial functions during homeostasis or pathogenesis in health/disease [6].

MicroRNAs (miRNAs) are endogenous, 22–25 nucleotide RNAs that play major regulatory roles in higher eukaryotes by targeting mRNAs for cleavage or translational repression. MiRNAs modulate the innate and adaptive immune responses to pathogens by affecting host immune cell differentiation and progression of diseases [7]. The clinical application of miRNAs as diagnostic or prognostic biomarkers has already been demonstrated in various types of cancers [8]. However, compared with their well-known role in cancer, the biological and diagnostic role of miRNAs in LTBI is still poorly understood. In the present study, we used U937 cell line as *in vitro* macrophage model, focused on the interaction between U937 macrophages and Mtb Hsp16.3, aiming to identify differentially expressed miRNAs in U937 macrophages. Our study intends to explore the potential function of miRNAs in the interaction of macrophages with Mtb Hsp16.3 and provide insights for investigating the role of macrophage homeostasis in LTBI.

## Methods

### Ethics statement and participants

The local ethics committee of the Beijing Tuberculosis and Thoracic Tumor Research Institute reviewed and approved the study. Written informed consent was obtained from participants before their enrollment in the study. Twenty clinical health care workers of Beijing Chest Hospital were recruited and all have history of close contact with active tuberculosis patient for more than two years. The four healthy controls were students of Suzhou Institute of Biomedical Engineering and Technology and had no history of contact with TB. Potential study participants were excluded if they had another infectious disease. The interferon gamma release assay (IGRA) (T-SPOT.TB, Oxford Immunuotec, Oxfordshire, UK) was used to distinguish the LTBI group from healthy control. Fourteen clinical health care worker participants were IGRA-positive and included as LTBI group while the four healthy control subjects were IGRA-negative.

### PBMC samples preparation

Peripheral venous blood (10 ml) was drawn from each subject and PBMC samples were isolated by density gradient separation using Lympholyte-H, immediately mixed with TRIzol (1 ml) and frozen at -80°C until RNA were extracted.

### Preparation of the IDLV and Infection

To obtain the Mtb Hsp16.3 expression vector pLVHsp-IRES-GFP, the encoding gene Rv2031c was amplified and cloned into the pLVX-IRES-GFP plasmid, and confirmed by sequencing. The Lenti-X HTX Packaging System (Integrase Deficient) (Clontech, Mountain View, CA, USA) was used to prepare the viral vector. The U937 cells were cultured in RPMI1640 medium (Gibco, Grand Island, NY, USA) containing 10% fetal bovine serum under 5% CO_2_ at 37°C, infected with viral IDLVs stock at 5:1 multiplicity of infection (MOI), refreshed with medium 6 h later and incubated for 64 h.

### Western blot analysis

Briefly, U937 cells were infected with IDLVs (Hsp/GFP), and control IDLVs (GFP), respectively. After 64 h, the cells were collected and then heated for 5 min at 95°C in 1 × protein loading buffer containing β-mercaptoethanol, and cell extracts were separated on 12% SDS-PAGE gel and transferred to PVDF membranes. The membranes were blocked with 5% skimmed milk-TBST, incubated with polyclonal rabbit anti-Mtb Hsp16.3 antibodies (prepared in our laboratory) and β-actin antibodies (Solarbio, Beijing, China), and finally visualized via reaction with a chemiluminescent HRP substrate (Millipore, Billerica, MA, USA).

### Microarray analyses of infected macrophages

KangCheng Biosciences (Shanghai, China) performed the miRNA profiling analysis. To determine the miRNA profiles for the two groups, total RNAs were purified using TRIzol (Invitrogen, Grand Island, NY, USA) and a miRNeasy mini kit (Qiagen, Shenzhen, China), labeled using the miRCURY™ Hy3™/Hy5™ Power labeling kit (Exiqon, Vedbaek, Denmark) and hybridized on the specific miRCURY™ LNA Array (v.18.0, Exiqon, Denmark) platform. The Exiqon miRCURY™ LNA Array (v.18.0) contains 2043 capture probes covering all human miRNAs, and could quantify genome-wide miRNA expression in the two groups. Images on the chip were scanned using an Axon GenePix 4000B microarray scanner (Axon Instruments, Foster City, CA, USA) and imported into GenePix Pro 6.0 software (Axon) for grid alignment and data extraction. MiRNAs with intensities >50 were used to calculate the normalization factor. Expression data were normalized using the median normalization. After normalization, average values of replicate spots of each miRNA were used for statistical analysis; differentially expressed miRNAs were identified through fold change filtering. Data are presented as means ± standard deviations. Analysis of variance tests or unpaired two-tailed Student t tests were used for statistical analysis. The data were regarded as significantly different at *P* < 0.05.

### Reverse transcription and quantitative real time-polymerase chain reaction (qRT-PCR) validation

The total RNAs were extracted from each two groups of infected U937 macrophages and PBMC samples using a mirVana™ miRNA Isolation Kit (Ambion, Austin, TX, USA). cDNA was reverse transcribed from total RNAs using the miRcute miRNA cDNA first-strand synthesis kit (Tiangen, Beijing), according to the manufacturer’s instructions. Using U6/5S RNA as the endogenous reference for normalization, qRT-PCR assays were performed on an ABI 7500 Real-Time PCR System (Applied Biosystems, Foster, CA, USA) using the miRcute miRNA qPCR Detection kit (SYBR Green) (Tiangen, Beijing, China). The experiments were conducted in triplicate.

### Pathway enrichment analyses

The predicted targets of the miRNAs were obtained from the TargetScan database [9], and the PITA database [10]. The intersections of the results obtained from these different software programs were regarded as the reliable target genes. The predicted miRNA target genes were analyzed for enriched KEGG pathways using the NCBI DAVID server ( http://david.abcc.ncifcrf.gov) with default settings [11].

## Results

### U937 Macrophages expressed Mtb Hsp16.3 and GFP, respectively

**Figure 1:**
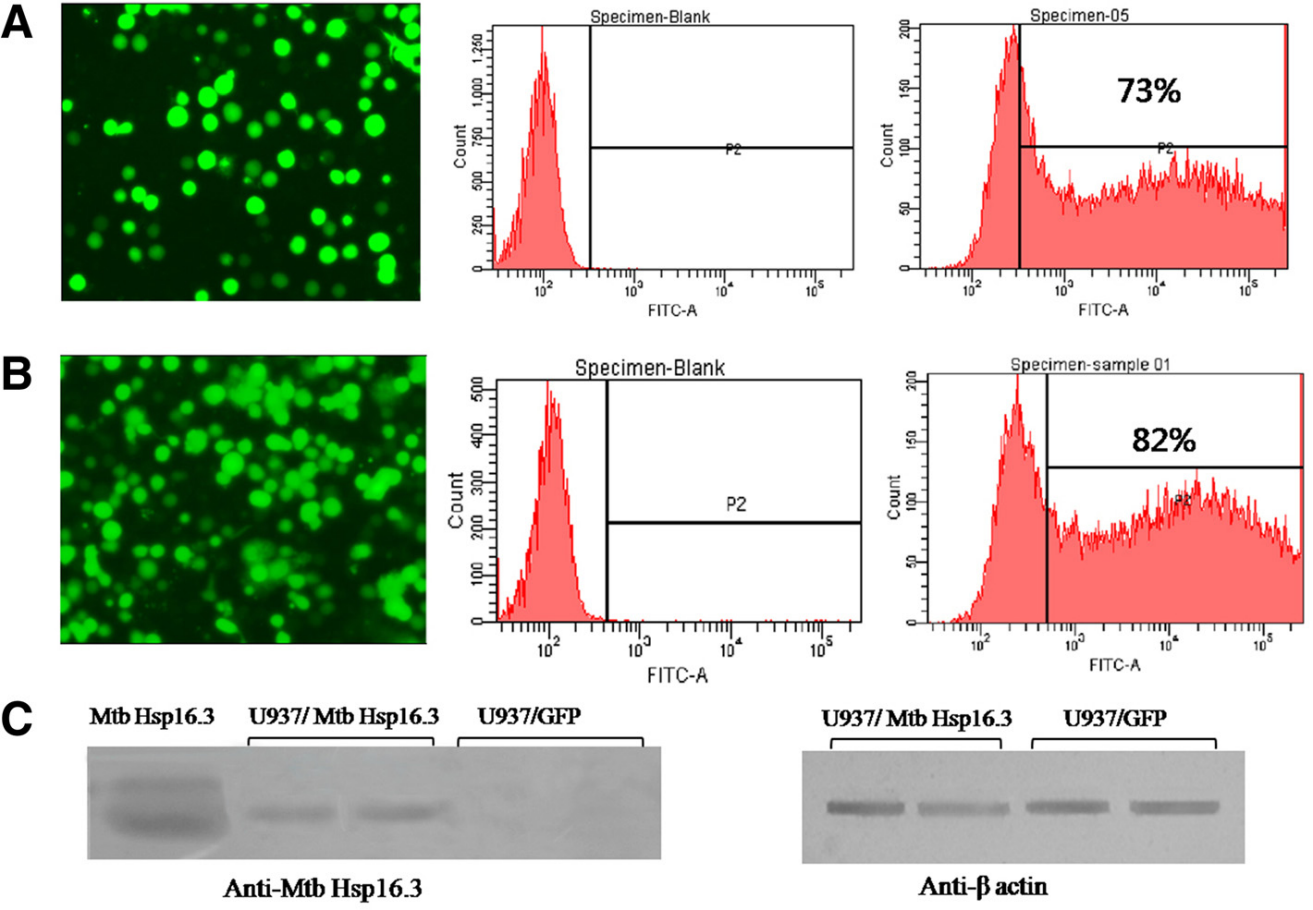
**The integrase-deficient lentivirus vector (IDLV) transfected U937 cells with high efficiency and the cells expressed Mtb Hsp16.3.** An IDLV delivered the transgene into U937 macrophages for instantaneous expression. The fluorescence microscopy and flow cytometry were used at 64 h after infection to detect GFP and analyse the transduction efficiency. **A**, the transduction efficiency of the test group of U937 cells (expressing Mtb Hsp16.3 and GFP) was 73%. **B**, the transduction efficiency of the control group (expressing GFP only) was 82%. **C**, western blot analysis with antibodies against Mtb Hsp16.3; β-actin was used as a loading control.

To reduce the risk of insertional mutagenesis in U937 cells, the IDLV system was used to produce non integrative lentiviral vectors , which delivered the transgene into U937 macrophages for instantaneous expression. After 64 h, the fluorescence microscopy was used to detect the GFP expression, and GFP-positive cells was quantified by the flow cytometry. It was shown that the transformation efficiency of the test group of U937 cells (expressing Mtb Hsp16.3) was 73% (Figure 1A), and that of the control group (expressing GFP) was 82% (Figure 1B). Furthermore, to validate the expression of Mtb Hsp16.3 protein in the cells, western blot analysis was performed using anti-Mtb Hsp16.3 and the results demonstrated that Mtb Hsp16.3 was strongly expressed in the test group of U937 cells (Figure 1C).

### Expression profiles of miRNAs in U937 cells from the test group and the control group

To determine the miRNA profiles for the two groups, the Exiqon miRCURY™ LNA Array was employed to perform the 2043 miRNAs assay (1898 human and 145 human viral miRNAs represented in the Sanger miRBase v18.0). After normalization and unsupervised filtering (see Methods), the obtained average values for each miRNA spot were used for statistical analysis. Comparing the data from the two groups (test/control) and using fold change filtering (upregulated more than 2-fold and downregulated less than 0.5-fold ), total of 149 differentially expressed miRNAs was identified, of which 60 were upregulated (Table 1) and 89 were downregulated (Table 2). The *P* values for these 149 miRNAs were less than 0.05 in the test groups compared to results for the control groups.

**Table 1:** Summary of upregulated miRNAs

**Name**	**Fold change**	***P *** **value**	**Chr. Loc.**	**Name**	**Fold change**	***P *** **value**	**Chr. Loc.**
hsa-miR-2355-3p	2.00	0.00162	2	hsa-miR-133b	4.30	0.00992	6
hsa-miR-451a	2.20	0.01085	17	hsa-miR-4664-3p	4.31	0.00022	8
hsa-miR-130b-3p	2.30	0.04627	22	hsa-miR-4431	4.35	0.00368	2
hsa-miR-486-5p	2.32	0.00208	8	hsa-miR-4804-3p	4.36	0.00023	5
hsa-miR-361-5p	2.33	0.04722	X	hsa-miR-18b-3p	4.62	0.00191	X
hsa-miR-3156-3p	2.50	0.00729	10	hsa-miR-675-3p	4.68	0.00028	11
hsa-miR-4728-3p	2.67	0.00029	17	hsa-miR-550b-3p	4.72	0.01382	7
hsa-miR-3191-5p	2.67	0.00020	19	hsa-miR-551a	4.75	0.00063	1
hsa-miR-296-5p	2.71	0.04951	20	hsa-miR-4685-3p	5.04	0.00090	10
hsa-miR-150-5p	2.85	0.00927	19	hsa-miR-23c	5.11	0.00081	X
hsa-miR-4540	2.86	0.01280	9	hsa-miR-5002-3p	5.14	0.00035	3
hsa-miR-4268	2.97	0.00969	2	hsa-miR-5689	5.33	0.00054	6
hsa-miR-1236	3.08	0.04877	6	hsa-miR-935	5.43	0.00187	19
hsa-miR-221-5p	3.16	0.03132	X	hsa-miR-374b-3p	5.79	5.4E-05	X
hsa-miR-3685	3.26	0.00356	12	hsa-miR-1255b-2-3p	5.83	0.00823	1
hsa-let-7d-3p	3.35	0.02153	9	hsa-miR-485-3p	6.00	0.00085	14
hsa-miR-3941	3.39	0.00646	10	hsa-miR-3938	6.03	0.00821	3
hsa-miR-498	3.47	0.0484	19	hsa-miR-374c-3p	6.04	0.00125	X
hsa-miR-548as-3p	3.49	0.00657	13	hsa-miR-377-5p	6.29	0.00024	14
hsa-miR-323a-3p	3.70	0.00350	14	hsa-miR-4324	6.39	0.00669	19
hsa-miR-550a-3p	3.71	0.00074	7	hsa-miR-4436b-5p	6.56	9.0E-05	2
hsa-miR-30e-3p	3.75	0.01335	Unknown	hsa-miR-1184	6.64	0.00266	X
hsa-miR-1273e	3.83	0.00201	Unknown	hsa-miR-5690	7.22	6.6E-05	6
hsa-miR-200b-3p	3.83	0.00148	1	hsa-miR-125b-2-3p	7.68	0.00145	21
hsa-miR-2113	4.02	0.01267	6	hsa-miR-4511	8.40	0.00580	15
hsa-miR-615-3p	4.03	0.00110	12	hsa-miR-548ao-3p	9.50	6.4E-05	8
hsa-miR-33b-5p	4.07	0.02481	17	hsa-miR-224-3p	13.23	0.00314	X
hsa-miR-147b	4.18	0.00080	15	hsa-miR-4278	14.61	9.4E-05	5
hsa-miR-7-2-3p	4.29	0.00900	15	hsa-miR-3680-5p	20.93	0.00474	16
hsa-miR-657	4.30	0.00035	17	hsa-miR-4678	31.50	0.00070	10

**Table 2:** Summary of downregulated miRNAs

**Name**	**Fold change**	***P *** **value**	**Chr. Loc.**	**Name**	**Fold change**	***P *** **value**	**Chr. Loc.**
hsa-let-7a-5p	0.038	1.1E-05	9	hsa-miR-3651	0.312	0.00422	9
hsa-miR-27a-3p	0.050	0.00148	19	hsa-miR-19a-3p	0.312	0.04552	13
hsa-miR-378c	0.053	0.00035	10	hsa-miR-106b-5p	0.315	0.00649	7
hsa-miR-3175	0.061	0.00039	15	hsa-miR-375	0.316	0.00187	2
hsa-miR-30a-5p	0.069	0.00115	6	hsa-miR-1973	0.326	0.00071	4
hsa-miR-374a-5p	0.078	0.00085	X	hsa-miR-4695-3p	0.331	5.7E-05	1
hsa-let-7f-5p	0.083	0.00068	9	hsa-miR-4279	0.335	0.00114	5
hsa-miR-424-5p	0.083	0.00112	X	hsa-miR-3182	0.342	0.00749	16
hsa-miR-16-5p	0.089	0.00715	13	hsa-miR-4454	0.342	0.00115	4
hsa-miR-181a-5p	0.106	0.04102	9	hsa-miR-4644	0.358	0.00413	6
hsa-miR-25-3p	0.129	0.00012	7	hsa-miR-197-3p	0.359	0.00547	1
hsa-miR-4653-3p	0.129	0.00054	7	hsa-miR-15a-5p	0.362	0.03027	13
hsa-miR-146a-5p	0.140	0.00239	5	hsa-miR-2115-3p	0.364	0.00016	3
hsa-miR-339-5p	0.146	0.00248	7	hsa-miR-937	0.365	0.00801	8
hsa-miR-5089	0.156	0.00179	17	hsa-miR-331-3p	0.374	0.00109	12
hsa-miR-493-5p	0.163	0.00619	14	hsa-miR-374b-5p	0.380	0.01720	X
hsa-miR-652-3p	0.164	0.00214	X	hsa-miR-1273 g-3p	0.382	0.00549	1
hsa-miR-21-5p	0.165	0.00059	17	hsa-miR-4668-5p	0.386	0.00013	9
hsa-miR-142-5p	0.175	0.00056	17	hsa-miR-20b-3p	0.390	0.01073	X
hsa-miR-3653	0.178	0.00117	22	hsa-miR-148a-3p	0.391	0.00075	7
hsa-miR-27b-3p	0.188	0.00133	9	hsa-miR-483-3p	0.392	1.4E-05	11
hsa-miR-299-3p	0.191	0.00112	14	hsa-miR-4450	0.393	0.00068	4
hsa-miR-1260a	0.193	7.5E-05	14	hsa-miR-93-5p	0.400	0.00736	7
hsa-miR-4445-5p	0.202	8.2E-05	3	hsa-miR-5684	0.405	0.00132	19
hsa-miR-301a-3p	0.207	0.00485	17	hsa-miR-4500	0.413	0.00962	13
hsa-miR-451b	0.210	0.00559	17	hsa-miR-3654	0.415	0.00400	7
hsa-miR-107	0.216	0.00010	10	hsa-miR-223-3p	0.416	0.00199	X
hsa-miR-196b-3p	0.226	0.00083	7	hsa-miR-3607-5p	0.421	0.00412	5
hsa-miR-5581-3p	0.229	9.8E-05	1	hsa-miR-93-3p	0.422	0.00129	7
hsa-miR-4417	0.230	0.00124	1	hsa-miR-24-3p	0.427	0.03788	9
hsa-miR-185-5p	0.239	0.01367	22	hsa-miR-365a-3p	0.433	0.00030	16
hsa-miR-1275	0.240	0.01379	6	hsa-miR-1260b	0.434	0.00267	11
hsa-miR-4636	0.241	0.00018	5	hsa-miR-4467	0.435	0.00152	7
hsa-miR-4787-5p	0.241	2.5E-05	3	hsa-miR-92b-3p	0.435	0.00053	1
hsa-miR-23b-3p	0.243	0.00758	9	hsa-miR-22-3p	0.436	0.01803	17
hsa-miR-30e-5p	0.244	0.04555	1	hsa-miR-1587	0.439	2.9E-05	X
hsa-miR-4286	0.254	3.0E-05	8	hsa-miR-142-3p	0.443	0.01233	17
hsa-miR-138-2-3p	0.256	0.00280	16	hsa-miR-26a-5p	0.448	0.00101	3
hsa-miR-29c-3p	0.260	0.01283	1	hsa-miR-644b-5p	0.458	0.01973	X
hsa-miR-4633-5p	0.261	0.00099	5	hsa-miR-15b-5p	0.460	0.03179	3
hsa-miR-7-5p	0.267	0.02246	15	hsa-miR-20b-5p	0.464	0.04709	X
hsa-miR-660-5p	0.280	0.00851	X	hsa-miR-4429	0.465	0.03150	2
hsa-miR-5000-3p	0.302	0.00034	2	hsa-miR-3646	0.470	0.00101	20
hsa-miR-30b-5p	0.303	0.00623	8	hsa-let-7d-5p	0.490	0.00531	9
hsa-miR-532-5p	0.309	0.00987	X				

### qRT-PCR validation of candidate miRNA expression level

**Figure 2:**
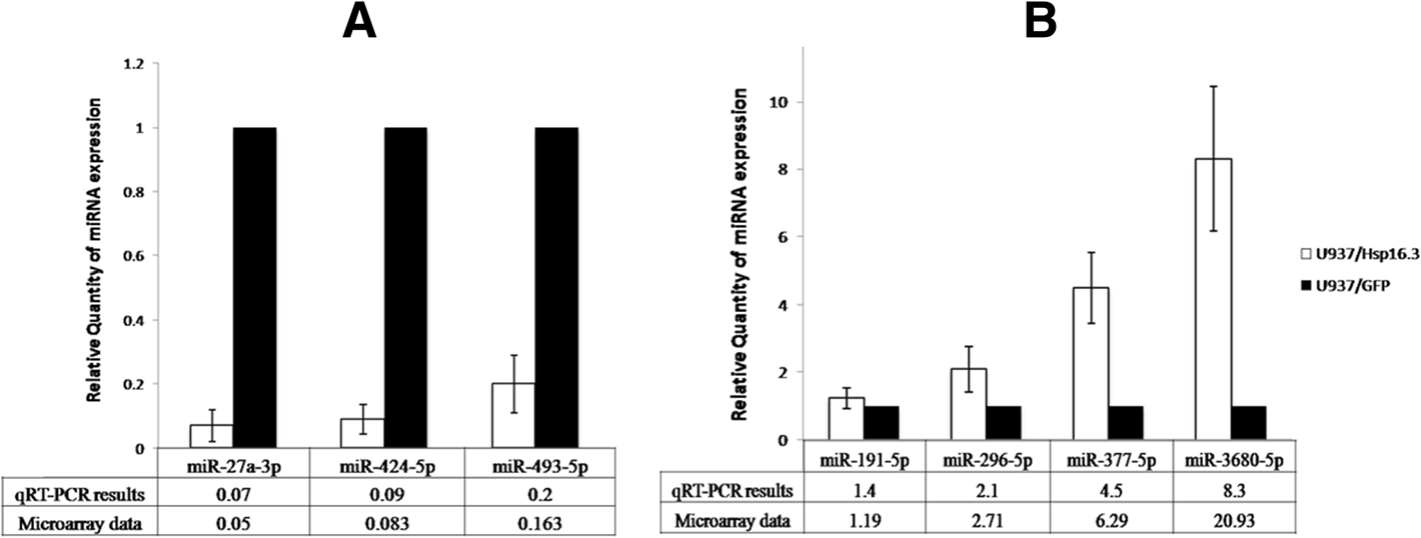
**Confirmation of miRNA expression profiles of the microarray by qPCR.** After normalization to 1 in the control group (U937/GFP), the relative expressions of selected downregulated miRNAs (miR-27a-3p, miR-424-5p, and miR-496-5p) in the test group are shown in **A**; the relative expressions of upregulated miRNAs (miR-296-5p, miR-377-5p, and miR-3680-5p), and unchanged miR-191-5p in the test group are shown in **B**.

**Figure 3:**
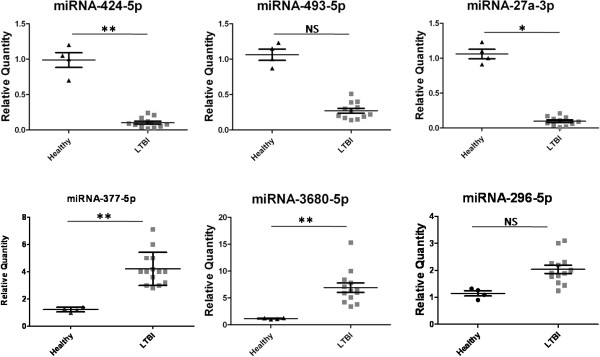
**qPCR validation of miRNA expression levels in samples from the latent tuberculosis infection (LTBI) group versus the healthy control group.** Relative expressions of miR-424-5p, miR-496-5p, miR-27a-3p, miR-377-5p, and miR-3680-5p in LTBI and healthy samples. Statistical analysis was performed using the unpaired t-test. **P < 0.01, *P < 0.05, NS: not significant.

To validate the microarray findings, seven miRNAs were selected for qRT-PCR analysis. The miR-424-5p (previous ID: miR-424), miR-493-5p (previous ID: miR-493*), and miR-296-5p were reported as potential to discriminate between latent TB and healthy by the previous study [12], the other four miRNAs (miR-27b-3p, miR-377-5p, miR-3680-5p, miR-191-5p) were randomly selected. As shown in Figure 2A, the respective level of downregulated miR-27a-3p, miR-424-5p, and miR-493-5p in qRT-PCR results largely reflected the altered patterns of these selected miRNAs observed in the microarray profiles. In parallel, the levels of upregulated miR-296-5p, miR-377-5p, miR-3680-5p, and unchanged miR-191-5p were similar to the chip results as well (Figure 2B). Furthermore, to evaluated the relative expression level of the six differentially expressed miRNAs in LTBI group and healthy control, 14 LTBI subjects and four healthy control individuals were recruited for the qRT-PCR assay (Additional file 1: Table S1). As shown in Figure 3, the results of four miRNAs (miR-424-5p, miR-27a-3p, miR-377-5p, miR-3680-5p) recapitulated the microarray data, and the other two miRNAs (miR-493-5p and miR-296-5p) were not significant differentially expressed.

### Bioinformatic exploratory analyses

Because only very small number of miRNA targets has been experimentally validated so far, we predicted the targets of these differentially expressed miRNAs using TargetScan and PITA software (Additional file 2: Table S2). The identified miRNAs were predicted to modulate 7044 target genes. We then used the NCBI DAVID server to identify the significantly enriched pathways involving the predicted target genes. As shown in Table 3, apart from cancer-associated pathways, the MAPK signaling, endocytosis, Wnt signaling, focal adhesion, axon guidance, and TGF-beta signaling pathways, which are related to differentiation, polarization, and versatility of macrophages, were significantly enriched. The results suggest that the miRNAs may regulate Mtb infection by affecting the development of immune cells.

**Table 3:** Enriched pathways involving the predicted target genes

**Pathway name**	**p value**
Pathways in cancer	5.60E-16
MAPK signaling pathway	1.70E-14
Endocytosis	6.90E-14
Neurotrophin signaling pathway	1.50E-13
Wnt signaling pathway	6.50E-13
Focal adhesion	7.60E-11
Axon guidance	1.10E-10
ErbB signaling pathway	7.10E-09
Glioma	5.80E-08
Basal cell carcinoma	6.20E-08
Long-term potentiation	6.30E-08
TGF-beta signaling pathway	9.10E-08
Regulation of actin cytoskeleton	1.10E-07
mTOR signaling pathway	3.70E-07
Adherens junction	1.30E-06
Chemokine signaling pathway	1.10E-05
Long-term depression	1.90E-05
T cell receptor signaling pathway	3.00E-05
Gap junction	5.60E-05
Fc gamma R-mediated phagocytosis	1.60E-04
B cell receptor signaling pathway	4.60E-04
GnRH signaling pathway	5.40E-04
Fc epsilon RI signaling pathway	7.60E-04
Phosphatidylinositol signaling system	1.50E-03
VEGF signaling pathway	1.50E-03
Vascular smooth muscle contraction	2.20E-03
SNARE interactions in vesicular transport	2.40E-03
ECM-receptor interaction	2.40E-03

## Discussion

The macrophage is the main replication niche of Mtb, despite the bactericidal characteristics and functions that this cell type normally has. The Mtb has evolved several strategies to reside and even replicate within the otherwise hostile environment of the macrophage, including the prevention of phagosome-lysosome fusion, inhibition of phagosomal maturation, and detoxification of the host’s stresses. Accordingly, the localization of Mtb inside the macrophage has been a matter of debate in recent years [13]. For a long time, an impermeable phagosome in the macrophage was thought to contain Mtb. However, recent evidence indicates that Mtb, as well as *M. leprae*, can escape its vacuole and reside in the host cell cytosol [14]. It is becoming clear that LTBI is not a static state with a homogenous population of non-replicating bacilli, but a constant endogenous Mtb reinfection process [15]. It is argued that both phagosomal maturation inhibition and escape from the phagosome are part of the survival strategies of Mtb. Owing to the lack of widely accepted animal or *in vitro* models to reflect the complex biology of LTBI, population-based studies are considered the best methods to investigate the intricate nature of LTBI. In some previous reports, human cell line U937 was used as *in vitro* model to investigate the molecular mechanism of Mtb during infection or persistence and its effect on the cell [16,17]. In this study, U937 cells expressing Hsp16.3 in the cytosol could partialy reflect the dynamic interplay of macrophages with dormant Mtb, which is necessary to prevent reactivation of the bacilli and development of active TB.

Indeed, some miRNAs that have been previously linked to carcinogenesis of different organs and tissues, such as miR-424-5p (previous ID: miR-424), miR-221-5p (previous ID: miR-221*), miR-675, miR-647, miR-125a-5p, miR-214-3p (previous ID: miR-214), miR-130b-3p (previous ID: miR-130b), miR-522-3p (previous ID: miR-522), and miR-16-5p (previous ID: miR-16) [18-21] were found to be up- or downregulated in our analysis. Forrest and colleagues [22] showed that induction of miR-424 (miR-424-5p) and miR-222 (miR-222-3p) promotes monocytic differentiation via combined regulation; both of these miRNAs were significantly downregulated in this analysis. Interestingly, miR-150-5p (previous ID: miR-150) has been shown to regulate the immune response and monocyte differentiation [23]; miR-150-5p was upregulated in our analysis. Conversely, miR-181a (miR-181a-5p) and miR-146a (miR-146a-5p), which have been proven to participate in the regulation of the adaptive immune responses, were 7- and 10-fold downregulated in our profiling data [24,25]. Furthermore, current research has demonstrated that miR-181a regulates inflammation responses in macrophages, and increased expression of miR-181a is strongly correlated with the expression of interleukin (IL)-1β, IL-6, and tumor necrosis factor alpha (TNFα) [26]. These results suggest that Hsp16.3 protein might be involved in blocking immunity against Mtb via miR-181a and miR-146a deregulation. In addition, Fu et al. demonstrated that miR-93*(miR-93-3p) was the most upregulated in active TB serum [27]; however, our analysis indicated that miR-93-3p was downregulated, making it a potential diagnostic marker to distinguish latent TB from active TB.

Although many target genes have been predicted by bioinformatic methods, the functions of most differentially expressed miRNAs remain unknown, and very few predicted target genes have been validated. More than half of the differentially expressed miRNAs did not find a target mRNA in either database; most of them were recently identified miRNAs. Bioinformatic exploratory provides a rapid analytic approach categorizing large amounts of genes into functionally related groups to thereby facilitate the uncovering of the biological content captured by transcriptomic profiling. KEGG pathway enrichment analyses further interpret the biological functions of these genes. The overrepresented pathways associated with glioma and basal cell carcinoma were enriched, which somewhat surprised. The formation of extra-pulmonary tuberculosis is caused by Mtb dissemination to other organs or tissue, such as the central nervous system (CNS) [28]. It is speculated that the occurrence of glioma might be related to tuberculoma in the CNS, and a tuberculoma-like granuloma is often misdiagnosed as a tumor [29]. This indicated that Mtb Hsp16.3 might be involved in carcinogenesis, which warrants further investigation.

Earlier studies, which used peripheral blood mononuclear cells (PBMCs) or whole blood cells to perform whole genome transcriptional profiling and miRNA profiling [27,30], described a number of candidate biomarkers that might function in active TB. Wang and collegues identified miRNAs that were differently expressed in latent TB versus healthy from the clinical PBMC samples [12], In present study, the microarray data and independent qRT-PCR results indicated that our *in vitro* model by used of U937 cells expressing Mtb Hsp16.3 protein has good repeatability. However, the weakness of the model is also obvious, it does not represent the real interaction of pathogen and host macrophage *in vivo*, it provided only mechanistic insights on the interaction between Mtb antigen and human cell line. Although the expressions of miR-424-5p (previous ID: miR-424), miR-27a-3p, miR-377-5p and miR-3680-5p were consistent in clinical PBMC samples, the small size of healthy controls weakened the statistical power. Our understanding the biology of latent tuberculosis as part of a broad range of responses that occur following infection with Mtb remains incomplete. Multiple factors are involved in this complex process. Herein, compared to previously studies, our experiments got more differentially expressed miRNAs since we focused on just whether the Mtb Hsp16.3 had great effects on the U937 macrophage cell. Furthermore, this model could also be used in the follow-up investigation of the miRNA candidates regulating the macrophage in chronic inflammatory response or other process correlated with LTBI.

## Conclusions

Using miRNA expression profiling, we identified 149 differentially expressed miRNAs and validated that the transcription patterns of some miRNAs were consistent with previous reports. Our data provide evidences for the underlying biological processes involved in LTBI via the interaction between U937 macrophages and the Mtb Hsp16.3 protein. These findings provide an improved understanding of the link between miRNA homeostasis and LTBI. Further characterization of the pathogenetic roles of specific miRNAs and deciphering of the miRNA-controlled signaling regulatory network may help to enhance diagnosis and prevention of LTBI.

### Supporting information

Microarray data submission for human arrays is MIAME-compliant. The chip data from this study have been deposited at NCBI Gene Expression Omnibus (GEO) database, and its accession number is GSE54630.

## Competing interests

The authors declare that they have no competing interests.

## Authors’ contributions

CZ and ZDZ conceived the study. QLM, FL, XYY, XML, and XZ carried out the experiments. QLM wrote the manuscript. All authors read and approved the final manuscript.

## Supplementary Material

Additional file 1: Table S1 Characteristics of latent TB infection and healthy control participants used for qRT-PCR. Click here for file

Additional file 2: Table S2 Target genes of differently expressed miRNAs. Click here for file
