# Supplementary material for: Identification of latent tuberculosis infection-related microRNAs in human U937 macrophages expressing Mycobacterium tuberculosis Hsp16.3
Source: BMC Microbiol. 2014 Feb 12;14:37. doi: 10.1186/1471-2180-14-37 (PMC3925440; doi:10.1186/1471-2180-14-37)
Supplement: Additional file 1: Table S1 — Characteristics of latent TB infection and healthy control participants used for qRT-PCR. [file 1471-2180-14-37-S1.docx]

| Characteristics of LTBI participants | | | | | | |
| --- | --- | --- | --- | --- | --- | --- |
| Gender (no. female/male) | | | | 15/5 | | |
| Age,mean (range) yr | | | | 32.65 ± 7.96 (22～45) | | |
| No. | Gender | Age | Contact with TB patient（year） | T.SPOT-  A antigen | T.SPOT-  B antigen | Result |
| 1 | female | 27 | 7 | 24 | 32 | positive |
| 2 | female | 33 | 8 | 108 | 20 | positive |
| 3 | male | 23 | 1 | 0 | 0 | negative |
| 4 | female | 32 | 2 | 0 | 43 | positive |
| 5 | female | 41 | 16 | 4 | 88 | positive |
| 6 | male | 44 | 25 | 36 | 0 | positive |
| 7 | female | 32 | 2 | 0 | 0 | negative |
| 8 | female | 44 | 21 | 100 | 72 | positive |
| 9 | female | 23 | 2 | 0 | 0 | negative |
| 10 | female | 23 | 3 | 0 | 0 | negative |
| 11 | male | 22 | 3 | 132 | 8 | positive |
| 12 | male | 23 | 4 | 16 | 28 | positive |
| 13 | female | 42 | 24 | 16 | 28 | positive |
| 14 | female | 38 | 19 | 60 | 8 | positive |
| 15 | female | 39 | 14 | 12 | 20 | positive |
| 16 | female | 45 | 21 | 36 | 16 | positive |
| 17 | female | 30 | 9 | 13 | 52 | positive |
| 18 | female | 30 | 13 | 32 | ~~4~~ | positive |
| 19 | male | 27 | 5 | 0 | 0 | negative |
| 20 | female | 35 | 15 | 0 | 0 | negative |

| Characteristics of LTBI participants | | | | | | |
| --- | --- | --- | --- | --- | --- | --- |
| Gender (no. female/male) | | | | 0/4 | | |
| Age,mean (range) yr | | | | 26.75 ± 2.22 (25～30) | | |
| No. | Gender | Age | Negative control | T.SPOT-A antigen | T.SPOT-B antigen | Result |
| 1 | male | 26 | 0 | 1 | 0 | negative |
| 2 | male | 26 | 0 | 2 | 0 | negative |
| 3 | male | 30 | 0 | 0 | 1 | negative |
| 4 | male | 25 | 0 | 3 | 0 | negative |
| Criteria standards: | | | | | | |
| 1、When the spots number of negative control were 0-5, the positive result should be: (spots number of A antigen or B antigen) － (spots number of negative control) ≥6 | | | | | | |
| 2、When the spots number of negative control≥6，the positive result should be: (spots number of A antigen or B antigen) ≥ 2× (spots number of negative control). | | | | | | |
